# Nonenzymatic Electrochemical Glutamate Sensor Using Copper Oxide Nanomaterials and Multiwall Carbon Nanotubes

**DOI:** 10.3390/bios13020237

**Published:** 2023-02-07

**Authors:** Md Younus Ali, Dorian Knight, Matiar M. R. Howlader

**Affiliations:** 1 Department of Electrical and Computer Engineering, McMaster University, 1280 Main Street West, Hamilton, ON L8S 4K1, Canada; 2 Department of Computing and Software, McMaster University, 1280 Main Street West, Hamilton, ON L8S 4K1, Canada

**Keywords:** glutamate biosensor, electrochemical sensing, nanomaterial, CuO-MWCNTs, nonenzymatic, voltammetry

## Abstract

Glutamate is an important neurotransmitter due to its critical role in physiological and pathological processes. While enzymatic electrochemical sensors can selectively detect glutamate, enzymes cause instability of the sensors, thus necessitating the development of enzyme-free glutamate sensors. In this paper, we developed an ultrahigh sensitive nonenzymatic electrochemical glutamate sensor by synthesizing copper oxide (CuO) nanostructures and physically mixing them with multiwall carbon nanotubes (MWCNTs) onto a screen-printed carbon electrode. We comprehensively investigated the sensing mechanism of glutamate; the optimized sensor showed irreversible oxidation of glutamate involving one electron and one proton, and a linear response from 20 μM to 200 μM at pH 7. The limit of detection and sensitivity of the sensor were about 17.5 μM and 8500 μA·mM^−1^·cm^−2^, respectively. The enhanced sensing performance is attributed to the synergetic electrochemical activities of CuO nanostructures and MWCNTs. The sensor detected glutamate in whole blood and urine and had minimal interference with common interferents, suggesting its potential for healthcare applications.

## 1. Introduction

Glutamate is the most abundant neurotransmitter in the central nervous system and plays an important role in different physiological processes, such as thinking and learning. It is a nonessential amino acid biomarker which can be synthesized adequately by the human body. However, excess quantities of glutamate can cause excitotoxicity, resulting in pathologies and disorders [1,2]. Glutamate-associated pathologies include neurodegenerative diseases, such as chronic pain [3,4], Alzheimer’s [5], Huntington’s disease [6], stroke [7] and cancer [8]. These pathologies can be continuously monitored through real-time measurements of glutamate levels in biofluids such as saliva, urine, and blood. Detecting glutamate in biofluids allows for early diagnosis of these diseases.

Existing methods to detect glutamate include conventional lab-based methods such as capillary electrophoresis [9], fluorescence detection [10], high performance liquid chromatography [11] and gas chromatography-mass spectroscopy [12]. While these methods offer low limits of detection (LOD) and high sensitivity of glutamate, they are bulky, expensive, time-consuming and require trained personnel to operate the equipment. On the other hand, electrochemical sensors can precisely detect biomarkers in biofluids with fast response, low LOD, and high sensitivity. Thus, these sensors are highly beneficial for real-time monitoring of biomarkers for early diagnosis of diseases. However, due to nonelectroactive nature of glutamate, it is challenging to detect glutamate at low potential range (e.g., cyclic voltammetry) and physiological conditions (e.g., pH) [13,14]. To address this issue, a significant amount of research has been done on enzymatic glutamate sensing, where glutamate oxidase (GluOx) or glutamate dehydrogenase (GLDH) is incorporated into the working electrode to catalyze the glutamate oxidation [13,15,16,17,18]. While enzymatic sensors are sought-after for their high selectivity, they often lack stability and are expensive [19].

Nonenzymatic electrochemical sensors are gaining remarkable attention for glutamate sensing due to their fast response, high stability and low cost, which can overcome the issues with enzymatic glutamate sensing. Different nonenzymatic sensing electrodes modified with nanocomposites and functional materials such as platinum-coated nickel nanowire arrays electrodes (Pt/NiNAEs) [20], nickel oxide (NiO) nanoparticles (NPs) modified glassy carbon electrodes (GCEs) (NiO/GCEs) [21], cobalt oxide nanosheets (Co_3_O_4_ NSs)/GCE [22] and copper oxide and gadolinium oxide (CuO.GdO NSs)/Nafion/GCEs [23], have been demonstrated for the detection of glutamate. The sensor based on NiNAE and Pt/NiNAEs [20] detected glutamate in a basic medium using the catalytic activity of nickel (Ni) nanowires (NWs) and offered good stability. However, the linear range (LR) of 500 µM to 8 mM is very high compared to the level of glutamate present in any biofluids in the human body [2,17], making it inappropriate for monitoring glutamate in real samples. Additionally, the mass fabrication of Ni NWs is not cost-effective, and the use of Pt NPs is expensive. The NiO/GCE sensor was reported in [21], which detected glutamate at a very high pH, using the catalytic activity of NiO with a 0.1 M NaOH electrolyte. The linear range and LOD of the sensor were higher than the levels of glutamate in biofluids. In terms of LOD and linear range, improved sensing performance was reported in [22,23] using CO_3_O_4_ NSs/GCEs and CuO.GdO NSs/Nafion/GCEs, respectively. However, the low sensitivity and poor selectivity of the sensor led to unreliable detection of glutamate. Furthermore, the use of GCEs impedes the development of tiny, integrated sensing systems. A mediator-free, non-enzymatic glutamate sensor was developed by Zeynaloo using a screen-printed carbon electrode (SPCE) modified with gold (Au) NPs and a glutamate binding protein (GluBP) [24]. The GluBP/AuNP/SPCE offered selective detection of glutamate with an LR of 0.1–0.8 µM, but the usage of Au NPs is costly, while the usage of the glutamate binding protein may cause instability in the sensor. The challenges of nonenzymatic glutamate sensors are high LODs, difficulty sensing with physiological conditions [20,21], and poor selectivity [20,21,22,23]. Furthermore, the glutamate sensing mechanism and the role of sensing environments and parameters, such as the effect of pH and scan rate, have not yet been clearly understood [20,21,22,23]. Therefore, there is more room to investigate the effects of the sensing environment and the nonenzymatic electrochemical sensing mechanism of glutamate.

Nanomaterials are widely used in sensing to achieve low LOD and high sensitivity due to their high surface-to-volume ratio, excellent catalytic activity, and adsorption capacity, as well as tunable electronic and electrochemical properties compared to their bulk counterparts [25]. Multiwall carbon nanotubes (MWCNTs) are widely used in the detection of numerous biomolecules, such as glucose [26], c-reactive protein [27], glutamate [28], and DNA [29], due to their catalytic activity, excellent conductivity, and high electron transfer efficiency [30,31,32]. Cu is a transition metal that is widely used in electronics [33,34], sensing [35], and many other applications [36] because of its high conductivity, low cost, and compatibility with various fabrication techniques compared to other metals such as gold (Au) and platinum (Pt). However, copper’s tendency to oxidize in non-inert conditions [37,38,39,40], hinders its widespread usage in sensing applications. On the other hand, copper oxide (cuprous and cupric oxide) nanomaterials have attracted much attention for sensing due to their low cost, stability, and superb catalytic and semiconducting properties [23,40,41,42,43,44]. While CuO nanomaterials are widely used in electrochemical sensing, CuO-modified bare electrodes do not show enough sensitivity to detect trace amounts of target analytes due to the high resistance among CuO nanoclusters and the low effective surface area of the bare electrodes [45]. These challenges can be addressed by combining the large surface area and heterogeneous catalytic activities of CuO nanomaterials [46] with the excellent electronic and electrochemical properties of carbon nanotubes (CNTs) [31,32].

In this work, we developed a low-cost, ultra-high sensitive disposable nonenzymatic electrochemical glutamate sensor by modifying a screen-printed carbon electrode (SPCE) with CuO-MWCNT nanocomposites. To the best of our knowledge, there is no report on CuO-MWCNTs/SPCE for the detection of glutamate. We fabricated CuO nanostructures using a wet chemical precipitation process followed by dispersing CuO and MWCNTs in deionized (DI) water. After that, we drop-casted the CuO-MWCNTs suspension on the SPCE. The modified electrode utilizes the synergetic electrochemical catalytic property of CuO-MWCNTs, resulting in enhanced sensitivity toward glutamate sensing. We investigated the sensing mechanism through the scan rate and pH-dependent analyses, as well as sensing performances, including LOD, sensitivity, and interference. Finally, we demonstrated the application of the sensor for glutamate detection in real samples using whole blood and urine.

## 2. Materials and Methods

### 2.1. Reagents and Preparation of Samples

Copper (II) chloride, sodium hydroxide, L-glutamic acid, uric acid, ascorbic acid, L-cysteine, D-glucose and potassium ferricyanide (III) were purchased from Sigma Aldrich Canada. MWCNTs (>95%, OD: 5–15 nm, length: ~50 μm, electrical conductivity: >100 S·cm^–1^) were purchased from U.S. Research Nanomaterials Inc. Potassium chloride (KCl) and sodium phosphate (mono and dibasic) were purchased from ACP Chemicals Inc. All of these chemicals were of analytical grade. A stock solution of 1 mM L-glutamic acid, HOOC−CH(NH_2_)−(CH_2_)_2_−COOH (147.13 g/mol) was prepared by dissolving 14.731 mg of glutamic acid into 100 mL of DI water. Next, 0.1 M KCl (8 mg/mL) was used as a supporting electrolyte. Different concentrations of glutamic acid were prepared by diluting a 1 mM stock solution of glutamic acid into the KCl solution such that the final concentration of KCl is 8 mg/mL. Similar processes were used for preparing glucose, ascorbic acid and uric acid samples for the interference analysis. To prepare phosphate buffer saline (PBS) of different pH (pH 5.5–8.5), appropriate amount of KCl, Na_2_HPO_4_ and NaH_2_PO_4_ were dissolved into DI water.

### 2.2. Apparatus

We used a PalmSens EmStat 3 potentiostat for all electrochemical measurements. The SPCE electrode (3 mm diameter), a platinum (Pt) wire counter electrode, and Ag/AgCl reference electrodes were purchased from CH Instruments Inc. USA. Deionized (DI) water of resistivity ≥18.3 MΩ·cm (ELGA Purelab Ultra) was used for preparing aqueous solutions. Next, 4 mL of the analyte was taken into an in-house 3D printed vial (Supplementary Material Figure S1) for electrochemical measurements. A JPS-9200 from JEOL was used to acquire X-ray photoelectron spectroscopy (XPS) spectra for the chemical analysis of the sensing electrodes. The Magnesium X-ray source with 12 kV and 15 mA was used for acquiring wide-scan (not shown) and narrow scan spectra with a binding energy resolution of 0.1 eV. High-resolution optical microscopy was performed using the Alicona Infinite Focus G5 3D Surface Measurement System (Alicona Manufacturing Inc., Bartlett, IL, USA). The surface topography and morphology were examined using the tapping mode of an Anton Parr ToscaTM 400 Atomic Force Microscopy (AFM) (Graz, Austria) and a scanning electron microscope (SEM, JEOL 7100F). The topography and image processing and data analysis of the scans were conducted using the ToscaTM software (Version 7.4.8341).

### 2.3. Synthesis of Copper Oxide Nanostructure

**Figure 1:**
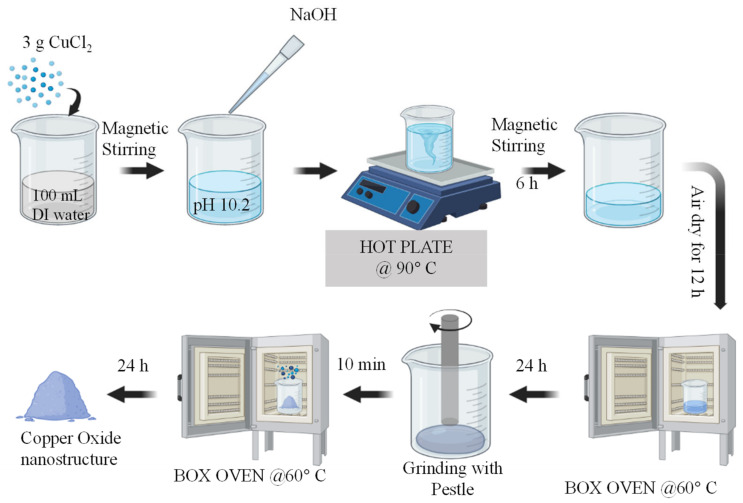
Wet chemical precipitation process for the synthesis of CuO nanostructures.

We synthesized copper oxide nanostructures using a wet chemical precipitation process [22]. First, 3 g of CuCl_2_ was dissolved in 100 mL of DI water, and NaOH was added dropwise to make the solution highly alkaline (pH 10.2). The solution was left at 90 °C with continuous stirring for 6 h. The beaker was then left to air dry for 12 h followed by heating in a box oven at 60 °C for 24 h. The dried CuO was taken out of the oven and ground to a fine CuO nanostructure. Finally, the CuO powder was put into the box oven for 24 h at 60 °C to promote further oxidation. This process is outlined in Figure 1.

CuCl_2 (s)_→Cu^2+^ _(aq)_ + 2Cl^−^_(aq)_

NaOH_(s)_→Na^+^ _(aq)_ + OH^−^ _(aq)_

Cu^2+^ _(aq)_ + 2Cl^−^_(aq)_ + 2Na^+^ _(aq)_ + 2OH^−^ _(aq)_→Cu(OH)_2 (aq)_ + 2NaCl _(aq)_

Cu(OH)_2 (aq)_→CuO_(s)_ + H_2_O

### 2.4. Preparation of CuO-MWCNTs/SPCE Electrode

**Figure 2:**
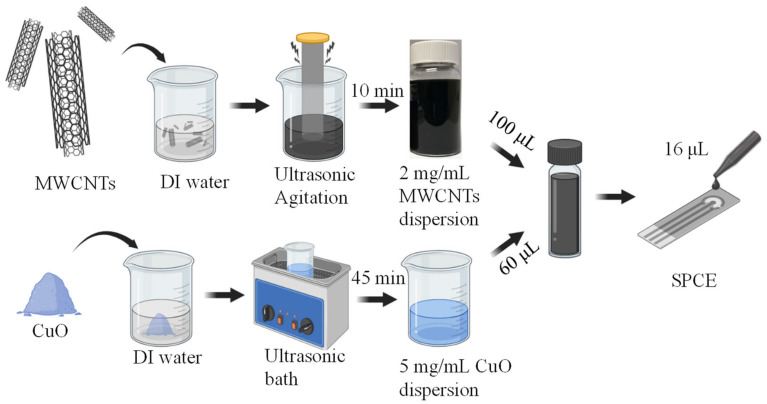
Schematic diagram of the preparation of the CuO−MWCNTs/SPCE-sensing electrode.

Figure 2 shows the schematic diagram of the preparation of the CuO-MWCNTs/SPCE-sensing electrode. The CuO nanostructure of 5 mg/mL was prepared by dispersing of 25 mg CuO into 5 mL DI water using ultrasonication for 45 min. MWCNTs of 2 mg/mL solution were prepared by dispersing 20 mg of MWCNTs into 10 mL DI water using a probe ultrasonic processor for 10 min. The CuO-MWCNTs composite was prepared by hand mixing the CuO dispersion with the MWCNTs dispersion. Finally, the CuO-MWCNT mixture was drop-casted into the unmodified SPCE electrode and then dried on a hot plate at 35 °C for 20 min. Physical mixing of 2 mg/mL MWCNTs and 5 mg/mL CuO with a 10:6 volume ratio and drop-casting 16 μL of CuO-MWCNTs on the SPCE gave the optimum sensing performance.

## 3. Results and Discussion

### 3.1. Characterization of CuO and CuO-MWCNT/SPCE Electrode

#### 3.1.1. Identification of Copper Oxides

**Figure 3:**
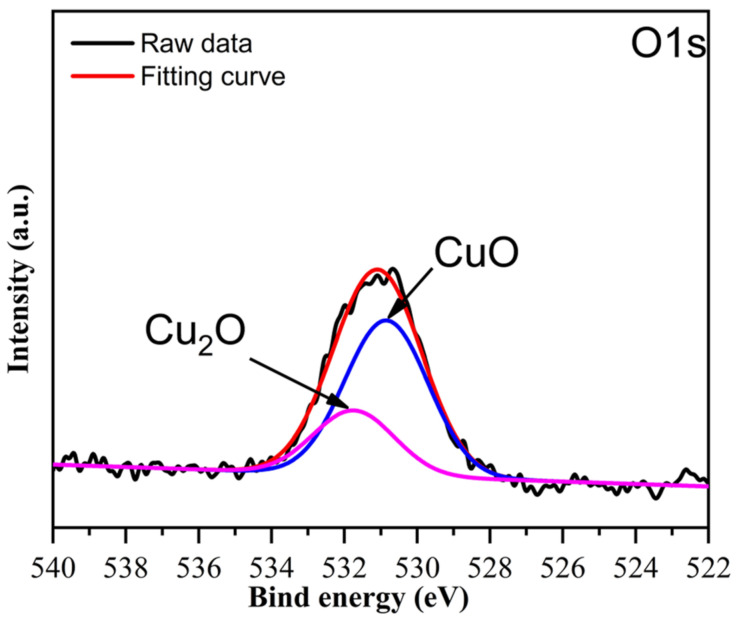
XPS O 1 s spectrum of the fabricated CuO nanostructures (peak separation and fitting model: Gauss+ Lorenz, R^2^ = 0.99).

We used an x-ray photoelectron spectroscope to investigate the chemical nature of the synthesized copper oxide nanostructures. The O 1 s spectra of the copper oxide is shown in Figure 3. The spectra possess two peaks at binding energies of 531.7 eV and 530.9 eV that represent Cu_2_O and CuO, respectively [47]. The Cu (II) oxide is dominant over Cu (I) oxide in the fabricated copper oxide nanostructures.

#### 3.1.2. Surface Morphology of Fabricated CuO and CuO-MWCNTs/SPCE

**Figure 4:**
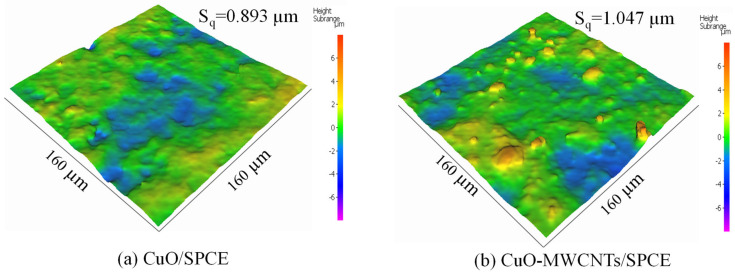
High resolution optical microscopic image of (**a**) CuO/SPCE and (**b**) CuO−MWCNTs/SPCE surface.

Surface morphology plays a significant role in sensing performance. High surface roughness offers a highly effective surface area, thus allowing for more analyte to interact with the electrode, resulting in high sensitivity. To examine the surface morphology of CuO/SPCE and CuO-MWCNTs on a large scale, we conducted optical microscopy with nanometer-scale resolution using the Alicona Infinite Focus G5 3D surface measurement system, as shown in Figure 4.

A considerable amount of surface coverage by CuO was observed (as shown in Figure 4a), with some valleys and peaks present on the electrode surface, likely due to the coagulation of CuO nanostructures. The root mean square (rms) height (Sq) of CuO/SPCE was 0.893. However, mixing MWCNTs with CuO improved the surface roughness (Sq = 1.047, as shown in Figure 4b), which is attributed to the formation of random islands by MWCNTs and CuO-MWCNTs.

**Figure 5:**
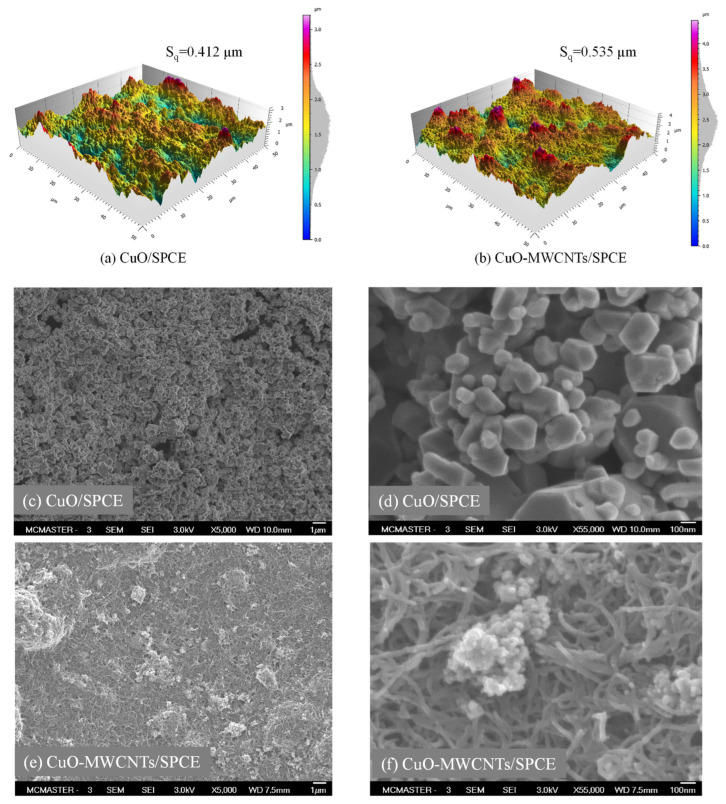
AFM surface images of (**a**) CuO/SPCE and (**b**) CuO-MWCNTs/SPCE surfaces; SEM images of (**c**,**d**) CuO/SPCE and (**e**,**f**) CuO-MWCNTs/SPCE surfaces.

We further examined the surface morphology and roughness of CuO/SPCE and CuO-MWCNTs/SPCE using AFM and SEM. The AFM image processing and data analysis were performed using ToscaTM software (Version-7.4.8341). Figure 5a,b shows the AFM images of the CuO/SPCE and CuO-MWCNTs/SPCE surfaces, respectively. Despite having high surface roughness, more island formation was observed on the CuO-MWCNTs electrode than on the CuO/SPCE electrode. The color bar indicates that for CuO/SPCE, the height of numerous sites ranges from 1 µm to 2 µm, while for CuO-MWCNTs/SPCE, the maximum heights fall between 2 µm to 3 µm. Figure 5c–f shows SEM images at different magnifications for different electrodes. From Figure 5c, it is observed that the surface of the SPCE electrode is homogeneously covered by CuO, possibly due to the small size of the CuO particles. The high magnification image (Figure 5d) reveals that the fabricated CuO is mainly composed of nanoparticles (NPs) with sizes ranging from 50 nanometers to several hundred nanometers. Figure 5e,f shows that CuO-MWCNTs uniformly cover the SPCE electrode surface, where CuO NPs are located within the MWCNTs network. However, numerous islands were observed due to the agglomeration of CuO NPs, resulting in high surface roughness as observed from AFM and optical microscope images.

### 3.2. Performance Optimization of Sensing Electrode

It is important to determine the optimal processing parameters for the fabrication of the sensing electrode to achieve maximum sensitivity. We used different durations of magnetic stirring (accumulation time) before voltammetry measurement to accumulate glutamate (Glu) on the CuO-MWCNTs/SPCE. The oxidation current increased with increasing accumulation time and reached a plateau for a stirring time of 10 min. Magnetic stirring facilitated the porous CuO-MWCNTs matrix to adsorb glutamate molecules through convection, resulting in enhanced sensitivity. In contrast, the oxidation current of glutamate was not improved with accumulation potentials from −0.5 V to 0.5 V. Therefore, we did not apply accumulation potentials for glutamate sensing. We stopped stirring after 10 min and conducted voltammetry measurements after 20 s of post-stirring. From the scan rate analysis, we found the optimal scan rate to be 60 mV/s for glutamate sensing.

### 3.3. Sensing Performance of the CuO-MWCNTs/SPCE Electrode

The concentrations of the supporting electrolytes and the range of sweeping potential have a significant influence on the redox potential of Cu and CuO [45,48,49,50]. The CV of the CuO-MWCNTs/SPCE electrode with different concentrations of KCl is shown in the Supplementary Figure S2. We observed optimum sensing performance at 8 mg/mL (0.1 M) KCl. Thus, in this work, 8 mg/mL (0.1 M) KCl is used as a supporting electrolyte for glutamate sensing. Since NaOH was used as a supporting electrolyte in several research works [20,21], we also examined different concentrations of sodium hydroxide (NaOH) as a supporting electrolyte for the detection of glutamate, using CuO-MWCNTs/SPCE as shown in Supplementary Figure S3. However, we did not notice any characteristic redox peak for glutamate using NaOH.

**Figure 6:**
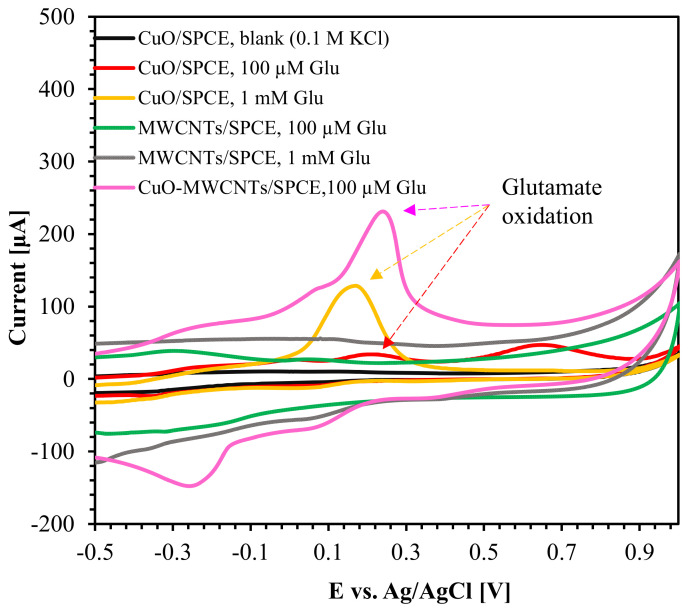
CV curves for different electrodes with 0.1 M KCl as the supporting electrolyte at a scan rate of 60 mV/s. The curves include: CuO/SPCE (black) in the absence of glutamate (Glu), CuO/SPCE (red) in 100 μM glutamate, CuO/SPCE (yellow) in 1 mM glutamate, MWCNTs/SPCE (green) in 100 μM glutamate, MWCNTs/SPCE (gray) in 1 mM glutamate, and CuO−MWCNTs/SPCE (purple) in 100 μM glutamate.

The oxidation of glutamate depends on the electrochemical activity of the CuO-MWCNTs sensing (working) electrode. To combine the catalytic activity of CuO with the electron transfer capability of MWCNTs, we investigated the individual roles of CuO and CuO-MWCNTs by modifying the SPCE with these materials. As shown in Figure 6, the cyclic voltammetry (CV) responses of MWCNTs/SPCE electrodes (green and gray lines) do not show any oxidation peak at 0.2 V to 0.4 V in 100 μM and 1 mM of glutamate. Therefore, MWCNTs alone do not have catalytic activity towards glutamate. In the blank solution (0.1 M KCl), CuO/SPCE does not show any oxidation peak at 0.2 V to 0.4 V). The oxidation peak of glutamate at 0.2 V to 0.4 V is only observed when the electrode contains CuO. Thus, CuO shows the catalytic activity towards glutamate. The effective surface area of the SPCE is low, and the CuO at the surface of the SPCE electrode only participates in the redox reaction [45]. As a result, the sensitivity of CuO/SPCE towards glutamate is low. However, for 100 μM glutamate, CuO-MWCNTs/SPCE offered about 130 μA of oxidation current, which is one order higher than that of CuO/SPCE. The blending of MWCNTs with CuO nanostructures creates numerous reaction sites and offers a high surface area compared to CuO/SPCE, as visualized from optical, AFM, and SEM images. Therefore, the use of MWCNTs enhances the electron transfer rate kinetics. The enhanced sensitivity of CuO-MWCNTs towards glutamate sensing is attributed to the synergistic electrochemical catalytic activity of MWCNTs and CuO. Since there is no reduction peak of glutamate, the electrochemical sensing of glutamate is an irreversible oxidation process.

**Figure 7:**
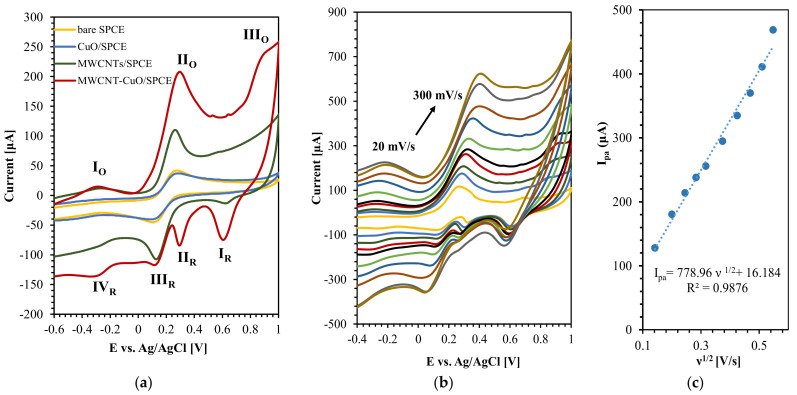
CV curves for (**a**) various electrodes at a scan rate of 60 mV/s, (**b**) CuO−MWCNTs/SPCE at different scan rates (20, 40, 60, 80, 100, 140, 180, 220, 260, and 300 mV/s), and (**c**) a plot of the square root of scan rate (*ν^1/2^*) versus peak oxidation current (*I_pa_*) in an aqueous solution of 5.0 mM K_3_[Fe(CN)_6_] with 0.1 M KCl.

To investigate the role of the effective surface area on the electrochemical activities of the CuO-MWCNTs/SPCE electrode, we used K_3_[Fe(CN)_6_] as a redox probe. In Figure 7a, the CV response of different sensing electrodes in the aqueous solution of 5 mM K_3_[Fe(CN)_6_] with 0.1 M KCl at a scan rate of 60 mV/s is shown. The bare SPCE and MWCNTs/SPCE sensing electrodes only show oxidation (II_o_) and reduction (III_R_) peaks of K_3_[Fe(CN)_6_] at about 0.3 V and 0.1 V, respectively. The I_O_ peak corresponds to Cu(0) oxidation since the synthesized CuO nanostructure contains Cu, Cu_2_O, CuO, and NaCl. In general, Cu(II) is not oxidized at potentials below 0.6 V, so III_O_ corresponds to the oxidation of Cu(II). I_R_ and II_R_ are the reduction peaks of Cu(III) and the IV_R_ peak represents the reduction peak of Cu(II) and Cu(I) [49]. The CuO-MWCNTs/SPCE-sensing electrode exhibited the highest redox current compared with other electrodes, which is attributed to the high electrical conductivity of MWCNTs and the combined electrochemical catalytic activity of CuO-MWCNTs. Figure 7b shows the CV response of the CuO-MWCNTs/SPCE electrode for different scan rates from 20 mV/s to 300 mV/s. All measurement were performed in triplicate.

The effective surface area of the CuO-MWCNTs/SPCE electrode can be calculated by using the Randles–Sevcik equation [51].

(1) $$I_{pa}=2.69\times 10^{5}AD^{1/2}n^{3/2}\nu^{1/2}c$$

where *I_pa_* is the peak current (A), *C* (mol/cm^3^) is the bulk concentration of the analyte, *A* is the effective surface area (cm^2^), *D* (cm^2^·s^−1^) is the diffusion coefficient, *n* is the number of electrons transferred and *ν* is the scan rate (V/s). K_3_[Fe(CN)_6_], *n* = 1, *D* = 6.67 × 10^−6^ cm^2^·s^−1^ and *C* = 5 × 10^−6^ mol/cm^3^ are used. By plotting the square root of the scan rate against the peak current, a linear relationship can be observed, as shown in Figure 7c. The slope of this curve is 780.54 × 10^−6^ A·s/V, which can then be used to calculate the effective surface area of the electrode. The effective surface area of the CuO-MWCNTs/SPCE electrode was found to be 0.224 cm^2^, which is three times higher than the physical area of the SPCE electrode (0.070 cm^2^).The high surface area of the CuO-MWCNTs/SPCE electrode can be validated by AFM and SEM images. This high effective surface area contributes to the enhanced sensitivity of the electrode towards the detection of glutamate.

### 3.4. Effect of Scan Rate on Glutamate Sensing

**Figure 8:**
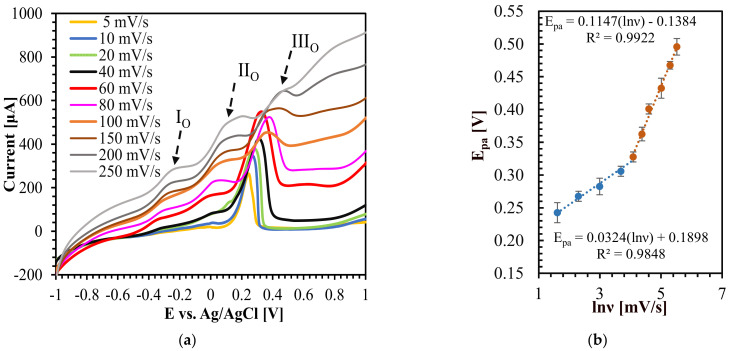
(**a**) LSV curves of the CuO−MWCNTs/SPCE electrode at different scan rates. (**b**) The dependency of the oxidation peak potential (*E_pa_*) of glutamate on the natural logarithm of the scan rate (*lnν*) in 0.1 M KCl and 400 µM glutamate.

A scan rate-dependent redox potential and current provides the charge transfer property of a sensing electrode. To reveal the electrochemical sensing mechanism of glutamate using the CuO-MWCNTs/SPCE electrode, we acquired the voltammetry responses at different scan rates in the blank (0.1 M KCl) (Supplementary Figure S3)) and with glutamate (Figure 8). In the presence of the KCl electrolyte, the anodic peak of Cu_2_O (III_O_) is a one-electron process and is dominant over CuO oxidation [45]. In the absence of glutamate, the III_O_ peak distorts at a scan rate of 40 mV/s and disappears at higher scan rates of ≥ 60 mV/s. At low scan rates, Cl^−^ ions not only move to the surface but also get enough time to penetrate deeper into the CuO-MWCNTs/SPCE electrode, while at high scan rates, Cl^−^ ion movement is limited to the surface of the electrode only.

Glutamate shows only oxidation; thus, we conducted linear sweep voltammetry (LSV) at different scan rates to investigate the charge transfer property of glutamate at the CuO-MWCNTs/SPCE electrode as shown in Figure 8. From Figure 8a, for the glutamate oxidation peak III_O_, peak oxidation potential increases with increasing the scan rate while the oxidation current increases with increasing scan rate until 60 mV/s and reduces at a higher scan rate (≥60 mV/s). A reduction in oxidation current is observed at high scan rates (≥60 mV/s), probably due to the adsorption of oxidized species of glutamate on the CuO-MWCNTs/SPCE electrode.

For an adsorption-controlled and irreversible electrode process, the oxidation potential can be expressed by the E. Laviron equation [52].

(2) $$E_{pa}=E_{0}+\left(\frac{RT}{\alpha nF}\right)ln\left(\frac{RTK^{0}}{anF}\right)+\left(\frac{RT}{\alpha nF}\right)ln\nu$$

where *E*_0_ is the standard electrode potential, *K*^0^ is the standard rate constant, *α* is the transfer coefficient, which is 0.5 for the irreversible process, and *n* is the number of electron transfers. The peak oxidation potential (*E_pa_*) vs. natural logarithm of scan rate (*lnν*) is shown in Figure 8b, which presents two linear relations between *E_pa_* and *lnν*.

For scan rate ≤60 mV/s, 


(3) $$E_{pa}=0.0324ln\nu +0.1892$$

For scan rate ≥60 mV/s, 


(4) $$E_{pa}=0.1147ln\nu -0.1304$$

The number of electrons involved in oxidation can be calculated from Equations (2)–(4), which are 0.0324 = *RT*/(*αn*_1_*F*) and 0.1147 = *RT*/(*αn*_2_*F*). For scan rate ≤60 mV/s, the number of electron transfer (*n_1_*) is two and for scan rate ≥60 mV/s, the number of electron transfer (*n_2_*) is one. Since Cu_2_O is oxidized at a low scan rate, therefore, for scan rate ≤60 mV/s, one electron comes from the oxidation of Cu_2_O [45], and another electron comes from the oxidation of glutamate. At a high scan rate ≥60 mV/s, no oxidation of Cu_2_O was observed (Figure S4), thus, at a high scan rate only glutamate is oxidized. The oxidation of glutamate at the CuO-MWCNTs/SPCE electrode involves one electron. The highest oxidation current was observed at a scan rate of 60 mV/s in the presence of glutamate (Figure 8a) and no oxidation peak (III_0_) of Cu_2_O was observed at a scan rate of 60 mV/s in the absence of glutamate. Therefore, a scan rate of 60 mV/s was used as the optimum value for glutamate sensing, as it offers high sensitivity and reduces the chance of false detection of glutamate.

### 3.5. Effect of pH on Glutamate Sensing

**Figure 9:**
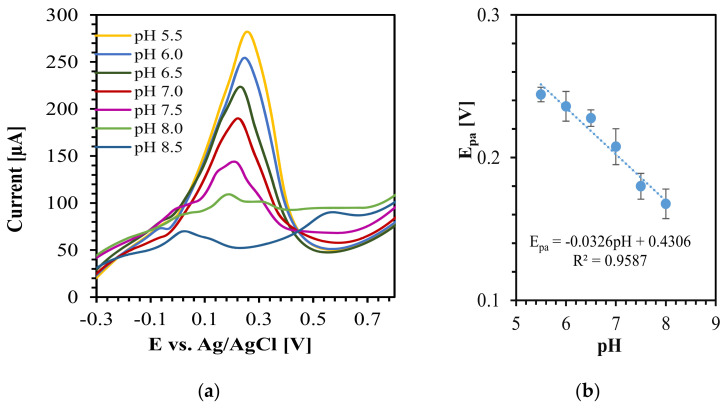
(**a**) LSV response of the CuO−MWCNTs/SPCE electrode in a 2 mM phosphate buffer (PB) with various pH levels in a 0.1 M KCl solution, and 1 mM glutamate at a scan rate of 60 mV/s. (**b**) The relationship between the oxidation peak potential (*E_pa_*) and *pH*.

We further investigated the number of electrons and protons (H^+^) involved in the oxidation of glutamate at the CuO-MWCNTs/SPCE electrode by observing the effect of pH (5.5–8.5) on glutamate oxidation, as shown in Figure 9. It is worth noting that a change in pH causes shifts in redox potential when H^+^ or OH^−^ is involved in the redox process. pH has a significant effect on glutamate sensing because it changes the ionic property of the glutamic acid (HOOC−CH (NH_2_)−(CH_2_)_2_−COOH).

According to Ref [53], at low pH (2.5 to 4.1), the NH_2_ group gains a proton, and the molecule charges to neutral zwitterion $-\mathrm{OOC}-\mathrm{CH}({\mathrm{NH}}_{3}^{+}-{\left({\mathrm{CH}}_{2}\right)}_{2}-\mathrm{COOH}$. At pH ≥ 4.1, it exists as a glutamate anion ($-\mathrm{OOC}-\mathrm{CH}\left({\mathrm{NH}}_{3}^{+}\right)-{\left({\mathrm{CH}}_{2}\right)}_{2}-\mathrm{COO}-$), which is prevailing in physiological pH range (7.35–7.45) and, at higher pH, the acid becomes a doubly-negative ion ($-\mathrm{OOC}-\mathrm{CH}\left(NH_{2}\right)-{\left(CH_{2}\right)}_{2}-\mathrm{COO}-$) [54]. Thus, a higher pH causes deprotonation of glutamic acid. In Figure 9, the decrease of oxidation potential and oxidation current with increasing pH indicates the involvement of protons in glutamate oxidation [51]. The decrease in oxidation current with increasing pH can be attributed to the deprotonation of glutamic acid. Since the glutamate ion is the dominant form of glutamic acid in the physiological pH range, we chose 0.1 M KCl as the supporting electrolyte (pH 7) for the detection of glutamate. If *m* and *n* are the numbers of protons and electrons, respectively, involved in the reaction process on the electrode, the oxidation potential can be expressed by [55]:


(5) $$E_{pa}=E_{0}-2.303\frac{mRT}{nF}pH$$

We plotted the oxidation potential (*E_pa_*) as a function of *pH* in Figure 9b. Since protons are a product in glutamate oxidation, therefore, according to Le Chatelier’s principle, increasing pH will increase the chance of proton formation by decreasing the oxidation potential [56]. From Figure 9b, the relation between oxidation potential and the *pH* can be expressed by the following linear regression equation:


(6) $$E_{pa}=-0.0326pH+0.4306\left(R^{2}=0.9587\right)$$

The ratio of *m* and *n* can be calculated from the slope of Equations (5) and (6), which is about 0.5. Thus, in the presence of glutamate, the oxidation process at the CuO-MWCNTs/SPCE electrode involves two electrons and one proton. In the absence of glutamate, the pH-dependent analysis confirmed that there is no shift in the peak oxidation potential of Cu_2_O, indicating that protons do not play a role in the oxidation of Cu_2_O (Figure S5). From the scan rate-dependent analysis (Figure 8), we confirmed that one electron comes from the oxidation of Cu_2_O, and one electron comes from the oxidation of glutamate. Therefore, at the CuO-MWCNTs/SPCE electrode, glutamate oxidation involves one electron and one proton.

**Scheme 1:**
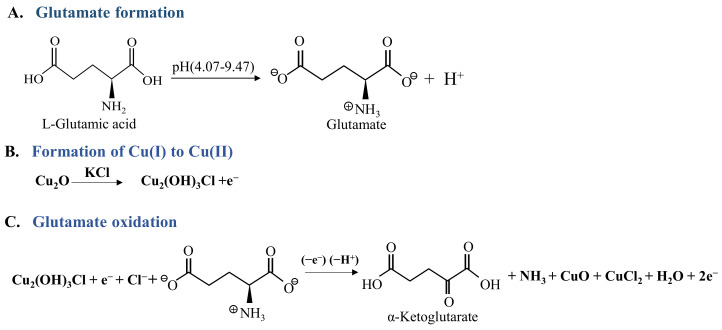
Sensing mechanism of glutamate at the CuO−MWCNTs/SPCE electrode.

In summary, we have schematically explained the sensing mechanism of glutamate, which involves three steps as shown in Scheme 1. First, L-glutamic acid converts to the glutamate anion in the pH range of 4.07–9.47 [53]. Second, Cu_2_O oxidation or CuO reduction produces a Cu-Cl product in KCl electrolyte [45]. Specifically, at pH 7, Cu_2_O oxidation in KCl may produce atacamite (Cu_2_(OH)_3_Cl) [57]. Finally, the atacamite may oxidize glutamate, resulting in α-Ketoglutarate [17], involving one electron and one proton as shown in Scheme 1.

### 3.6. Voltammetric Determination of Glutamate

**Figure 10:**
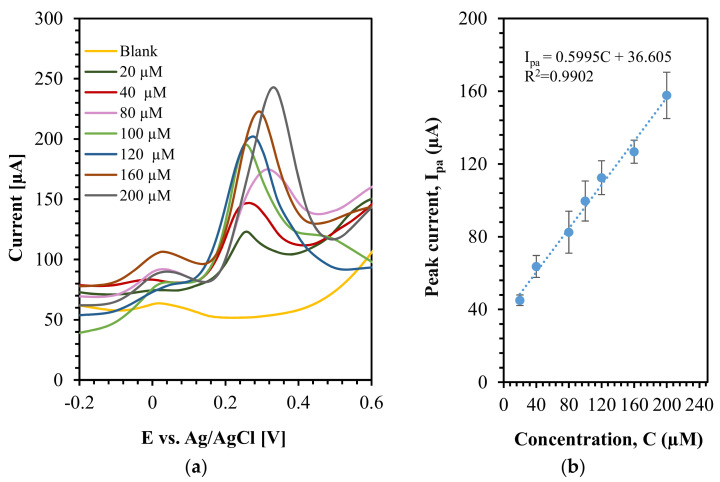
(**a**) LSV curves of the CuO−MWCNTs/SPCE electrode in 0.1 M KCl with different concentrations of glutamate at a scan rate of 60 mVs^−1^ with magnetic stirring for 10 min, and (**b**) calibration curve (*I_pa_* vs. concentration) of glutamate.

Figure 10a shows the LSV of the CuO-MWCNTs/SPCE-sensing electrode at different concentrations of glutamate with 0.1 M KCl at a scan rate of 60 mV/s. Figure 10b represents the calibration curve of glutamate for the concentration (*C*) ranges of 20–200 μM with a standard deviation of triplet measurements.

For the linear range (LR) of 20–200 µM, the linear regression equation is as follows:


(7) $$I_{pa}\left(\mu A\right)=0.5995C\left(\mu M\right)+36.605(R^{2}=0.9902)$$

We calculated the LOD of the sensor by using the following equation:

*LOD* = 3 *S_y_*/*m* (8)

where *S_y_* is the standard deviation of the response and *m* is the slope of the calibration curve. The estimated LOD was 17.5 µM. The physical area of the SPCE was 0.070 cm^2^. Therefore, the sensitivity of the sensor was about 8.5 µA·μM^−1^·cm^−2^ (8500 μA·mM^−1^·cm^−2^ or 8.5 A·M^−1^·cm^−2^). The catalytic properties of CuO nanostructures, high conductivity of MWCNTs and high effective surface area of the CuO-MWCNTs composite resulted in an ultra-high sensitive glutamate sensor. Table 1 shows the comparison of our work with recent nanomaterial-based nonenzymatic electrochemical sensors for the detection of glutamate. The LR and LOD of the developed sensor in this work are suitable for detecting glutamate in plasma, whole blood, and urine [2,17]. While the LODs in references [22,23] are lower than that of this work, those sensors showed low sensitivity and are prone to several interferences. In addition, the sensing approaches of references [22,23] are different from this work, resulting in difficulty comparing the sensing performances.

### 3.7. Interference Studies

**Figure 11:**
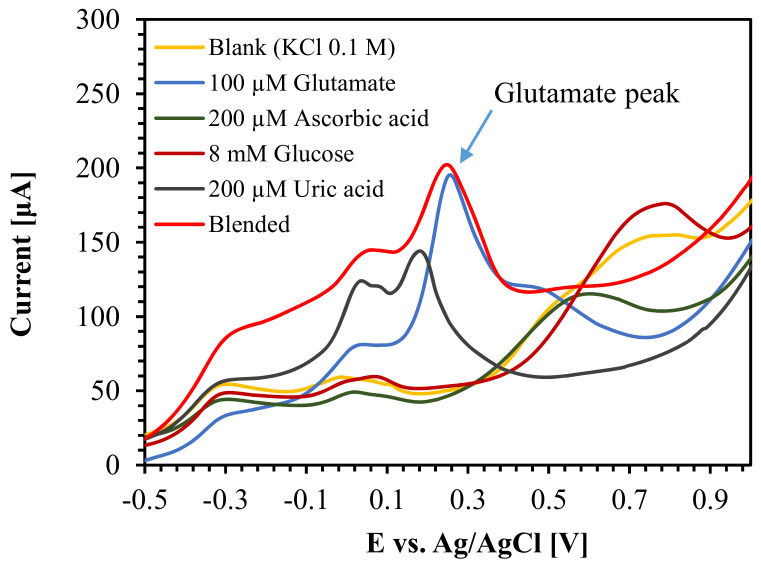
LSV curves for the CuO−MWCNTs/SPCE electrode in 0.1 M KCl with a scan rate of 60 mV/s, magnetic stirring for 10 min, and the following concentrations of substances: 100 µM glutamate, 200 µM ascorbic acid, 8 mM glucose, 200 µM uric acid, and 200 µM L−cysteine.

One of the challenges of nonenzymatic electrochemical sensors is the selective detection of target analytes. Body fluids, for example, blood, urine, and saliva, are complex matrices that consist of several biomarkers and analytes such as ascorbic acid, uric acid, glucose and glutamate. To investigate the selectivity of the CuO-MWCNTs/SPCE-sensing electrode towards glutamate, LSV was performed in the presence of those interferences and is presented in Figure 11. The electrode shows sensitivity towards uric acid; however, there was a considerable amount of potential difference in the oxidation peak of glutamate and uric acid. The blended curve represents the LSV response of a sample containing 100 µM glutamate, 200 µM ascorbic acid, 8 mM glucose, and 200 µM uric acid. The oxidation current of glutamate in the blended solution was a little bit higher than that of the solution containing glutamate alone. Therefore, the CuO-MWCNTs/SPCE electrode is reasonably selective towards glutamate.

### 3.8. Real Sample Analysis

**Figure 12:**
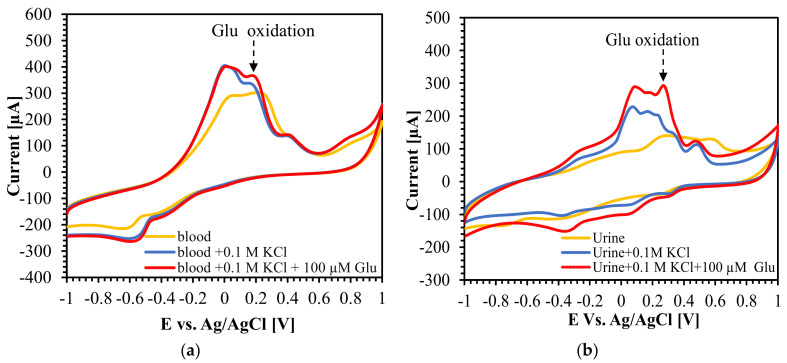
CV curves for the CuO−MWCNTs/SPCE electrode in (**a**) whole blood, and (**b**) urine at a scan rate of 60 mV/s with magnetic stirring for 10 min.

To examine the application feasibility of the CuO-MWCNTs/SPCE electrode in real-world samples, we detected glutamate in whole blood and urine as shown in Figure 12a,b. First, we detected glutamate in whole blood and urine without adding KCl electrolyte. Since the body fluids contain KCl, the sensor shows a glutamate peak without externally added KCl (yellow line). We observed a high oxidation current of glutamate in 0.1 M KCl (blue line). Finally, we added 100 µM glutamate using the standard addition method. The addition of glutamate in the sample increased the glutamate oxidation current in both whole blood and urine samples as shown in Figure 12a,b. Though the interference was observed in the CV response of whole blood and urine, the use of the peak separation method or principle component analysis [58] using machine learning could be useful to quantify the glutamate level in body fluids.

### 3.9. Effect of Temperature

**Figure 13:**
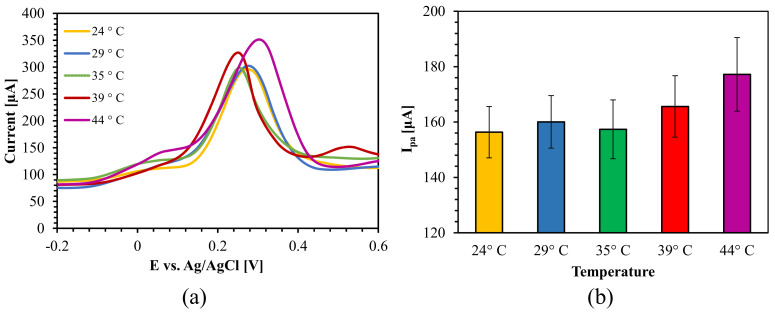
(**a**) LSV curves of the CuO−MWCNTs/SPCE electrode in 0.1 M KCl with 200 μM glutamate at different temperatures with magnetic stirring for 10 min at a scan rate of 60 mVs^−1^, and (**b**) bar plot of average peak oxidation current with a standard deviation of triplet measurements.

The effect of temperature on the analyte was examined by heating the analyte at different temperatures, followed by LSV, as shown in Figure 13. We found that the LSV response was almost the same until the analyte temperature reached 39 °C, where an increased oxidation current was observed. The current increased significantly when the temperature was ≥ 44 °C. This is probably due to the fact that at high temperatures, the mobility of ions in the analyte is high, resulting in more glutamate interacting with the electrode than at low temperatures. Additionally, the high oxidation current can be attributed to the high conductivity of CuO at high temperatures.

### 3.10. Repeatability and Reproducibility Studies

To examine the reproducibility of the sensor, we performed a recovery test of glu-tamate in 0.1 M KCl using LSV with optimum sensing parameters. We prepared a 1 mM stock and diluted it in 0.1 M KCl appropriately to prepare different concentrations of glutamate. The measured oxidation peak current (*Ipa*) was used in the calibration Equation (7) to determine the glutamate concentration (C). Table 2 shows that the sensor exhibited good recovery of glutamate from 92% to 112%.

## 4. Conclusions

In this work, we fabricated an ultra-high sensitive nonenzymatic electrochemical glutamate sensor. The sensing electrode was made by drop-casting a CuO and the MWCNTs nanocomposite onto an SPCE. To synthesize the CuO nanostructures, we used a wet chemical precipitation process. We optimized the sensing performance by changing the CuO and MWCNTs ratio, accumulation time, KCl electrolyte concentration, and scan rate. The physical mixing of 2 mg/mL MWCNTs and 5 mg/mL CuO with a 10:6 volume ratio gave the optimal performance. The KCl concentration-dependent analysis showed that 0.1 M KCl as a supporting electrolyte provided the best sensitivity. Magnetic stirring with the optimized value of 10 min increased the sensitivity due to the convection of glutamate towards the CuO-MWCNTs/SPCE electrode. The use of potassium ferricyanide as a redox probe showed that the CuO-MWCNTs/SPCE electrode had a very high effective surface area (0.224 cm^2^) which was three times higher than the physical area of the SPCE (0.070 cm^2^). Scan rate-dependent analysis in the absence and presence of glutamate with 0.1 M KCl solution confirmed that 60 mV/s is the optimum scan rate for high sensitivity and low false positive detection of glutamate. The Cu_2_O facilitated the oxidation of glutamate, and the MWCNTs enhanced the electron transfer rate. The scan rate and pH-dependent analysis demonstrated that the oxidation of glutamate is an irreversible process that involves one electron and one proton. At the optimum conditions, the sensor offered a linear response in the range of 20–200 µM of glutamate with a LOD of 17.5 µM. The sensor exhibited an ultra-high sensitivity of about 8500 μA·mM^−1^·cm^−2^, which is attributed to the synergetic electrochemical activities of CuO nanostructures and MWCNTs. The sensor showed good selectivity towards glutamate in the presence of common interference species in bodily fluids. The sensor detected glutamate in whole blood and urine and is thus promising for healthcare applications.

## Figures and Tables

**Table 1:** Comparison of recently developed nonenzymatic electrochemical sensors for glutamate detection.

Electrode	Detection Method	LOD (µM)	LR (µM)	Sensitivity (μA·mM^−1^·cm^−2^)	Electrolyte	Refs.
Pt/NiNAE	Amperometric	83	500–8000	96	1 M NaOH	[20]
NiNAE	Amperometric	135	500–8000	65	1 M NaOH	[20]
NiO/GCE	Amperometric	272	1000–8000	11	0.1 M NaOH	[21]
Co_3_O_4_-NS/GCE	I-V	10 × 10^−6^	10 × 10^−4^–10^5^	9.5 × 10^−2^	0.1 M PBS, pH 7	[22]
CuO.GdO NSs/Nafion/GCE	I-V	166 × 10^−6^	166 × 10^−6^–100 × 10^3^	0.567	pH 7 buffer	[23]
GluBP/AuNP/SPCE	CV	0.15	0.1–0.8		0.05 M PB, pH 7.4	[24]
CuO-MWCNTs/SPCE	LSV	17.5	20–200	8500	0.1 M KCl	This Work

**Acronyms**: Pt/NiNAE = platinum/Ni nanowire array electrode, NiO/GCE = nickel oxide/glassy carbon electrode, Co_3_O_4_-NS/GCE = Co_3_O_4_ nanosheets/glassy carbon electrode, CuO.GdO NSs/Nafion/GCE = CuO.GdO nanospikes/Nafion/GCE, GluBP/AuNP/SPCE = glutamate binding protein/gold nanoparticle/screen-printed carbon electrode. CuO-MWCNTs/SPCE = CuO-multiwall carbon nanotubes/screen-printed carbon electrode.

**Table 2:** The recovery test results for glutamate using the CuO-MWCNTs/SPCE electrode at a concentration of 0.1 M KCl.

Added (μM)	Found (μM) ^a^	Recovery (%)
40 μM	36.8	92
60 μM	67.38	112
100 μM	96.29	96
140 μM	142.44	101

^a^ Average values calculated from triplet measurements.

## Data Availability

The experimental data are available on request to corresponding author.

## Supplementary Materials

The following supporting information can be downloaded at: https://www.mdpi.com/article/10.3390/bios13020237/s1. Figure S1: Experimental setup. Working electrode (WE), counter electrode (CE), and reference electrode (RE). Figure S2: Effect of KCl electrolyte concentration on glutamate sensing. Figure S3: Effect of NaOH electrolyte concentration on glutamate sensing. Figure S4: CV curve of the CuO-MWCNTs/SPCE electrode at different scan rates in blank (0.1 M KCl) solution. Figure S5: LSV curves of the CuO-MWCNTs/SPCE electrode in 2 mM phosphate buffer (PB) pH from 5.5 to 7.5 and 0.1M KCl at a scan rate of 60 mV/s.
