# Supplementary material for: Nonenzymatic Electrochemical Glutamate Sensor Using Copper Oxide Nanomaterials and Multiwall Carbon Nanotubes
Source: Biosensors (Basel). 2023 Feb 7;13(2):237. doi: 10.3390/bios13020237 (PMC9954524; doi:10.3390/bios13020237)
Supplement: Supplementary file 1 [file biosensors-13-00237-s001.zip › biosensors-2152885-supplementary.pdf]

Supplementary Materials

Nonenzymatic Electrochemical Glutamate Sensor Using Copper Oxide Nanomaterials and Multiwall Carbon Nanotubes

Md Younus Ali [1], Dorian Knight [2] and Matiar M. R. Howlader [1,*]

[1] Department of Electrical and Computer Engineering, McMaster University, 1280 Main Street West, Hamilton, ON L8S 4K1, Canada
[2] Department of computing and software, McMaster University, 1280 Main Street West, Hamilton, ON L8S 4K1, Canada
* Correspondence: howladm@mcmaster.ca

The experimental set up for electrochemical sensing (voltammetry) of glutamate is shown in Figure S1.

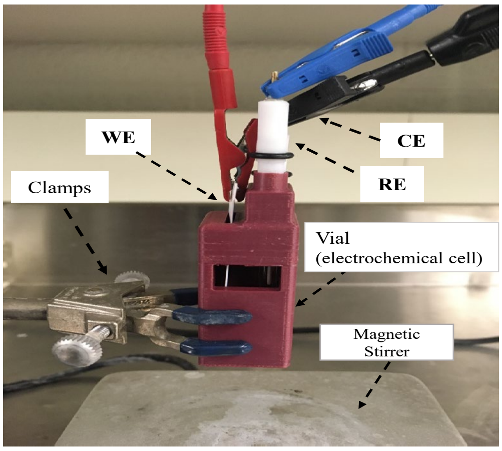

Figure S1. Experimental setup. Working electrode (WE), Counter electrode (CE), Reference electrode (RE).

Potassium chloride (KCl) was used as supporting electrolyte for the electrochemical sensing of glutamate using CuO-MWCNTs/SPCE. The concentration of KCl plays a crucial role in the sensing of glutamate, as shown in Figure S2. If the concentration of KCl is too high (>12 mg/mL), Cu_2O is highly oxidized at about 0 V, and no further oxidation is observed at 0.2-0.4 V, which is the oxidation potential of glutamate. If the KCl concentration is too low (<6 mg/mL), no characteristic oxidation peak of glutamate is observed, likely due to the high resistivity of the solution, which is not favorable for the oxidation of glutamate. Thus, in this work, 8 mg/mL (0.1 M) KCl was used as the supporting electrolyte.

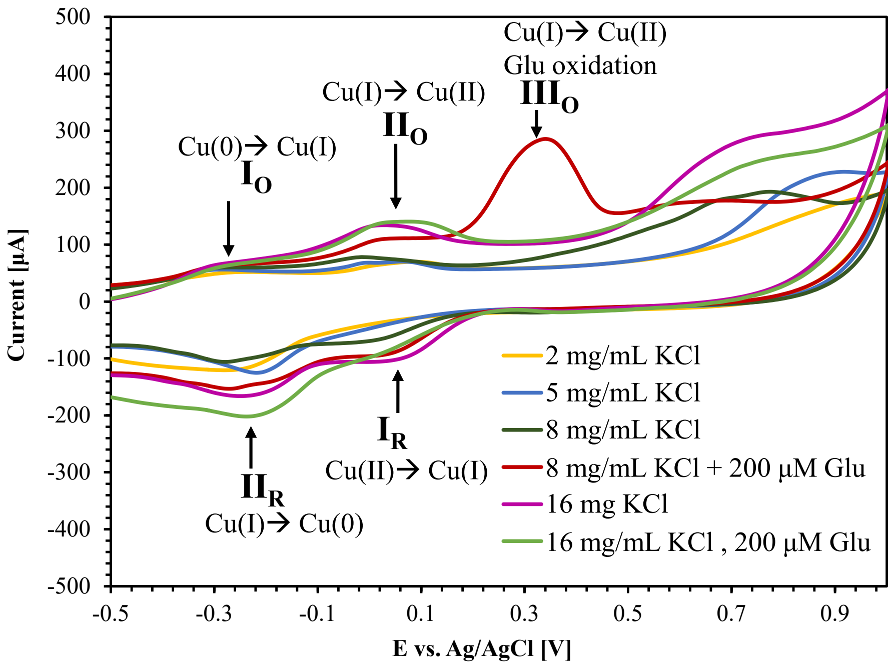

Figure S2. Effect of KCl electrolyte concentration on glutamate sensing.

Copper thin film based electrode was reported for sensing glucose at basic medium. Therefore, we examined different concentration of sodium hydroxide (NaOH) as supporting electrolyte for the detection of glutamate using CuO-MWCNTs/SPCE. However, we found that CuO-MWCNTs/SPCE did not show any characteristics peak for glutamate as shown in Figure S3. Using 0.1M NaOH, we found almost same CV response in the presence and absence of glutamate. However, the oxidation peak current of Cu (I) to Cu (II) at about 0 V depends on the concentration of NaOH as shown in the figure.

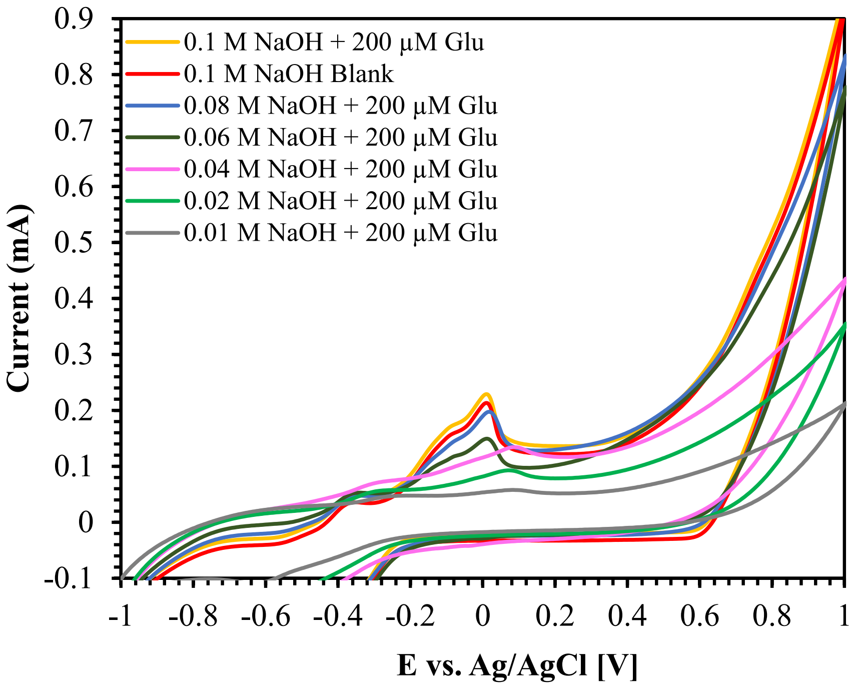

Figure S3. Effect of NaOH electrolyte concentration on glutamate sensing.

Figure S4 shows the CV response of CuO-MWCNTs/SPCE for different scan rates in the absence of glutamate in 0.1 M KCl. In the figure, I$_o$ denotes the oxidation of Cu, and II$_O$, III$_O$ denotes the oxidation of Cu_2O. For low scan rates (≤20 mV/s), the IIIO peak current

increases with increasing scan rate, and the oxidation potential shifts with the scan rate. Increasing oxidation current and shifting of oxidation potential with increasing scan rate indicate a quasi-reversible anodic peak III$_O$ in 0.1 M KCl for scan rates ≤20 mV/s. From Figure S4, it is also observed that the Cu_2O oxidation peak (III$_O$) distorts at a scan rate of 40 mV/s and disappears at higher scan rates (≥60 mV/s).

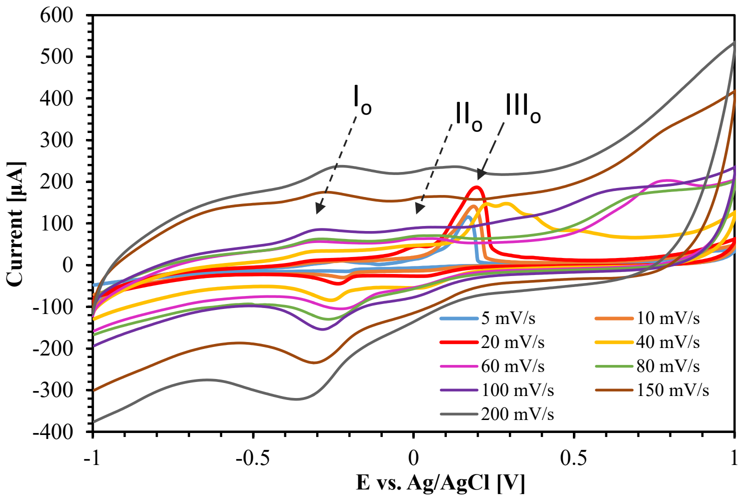

Figure S4. CV curve of CuO−MWCNTs/SPCE electrode at different scan rate in blank (0.1 M KCl) solution.

pH has a significant effect on the redox current and potential. To examine the effect of pH on Cu_2O oxidation, pH-dependent analysis was performed in the absence of glutamate. Figure S5 shows the LSV response of the CuO-MWCNTs/SPCE electrode at different pH levels in the blank solution containing 0.1 M KCl and 2 mM phosphate buffer. Since the oxidation potential is almost fixed at about 0.2 V and does not depend on pH, no proton is involved in the oxidation of Cu_2O.

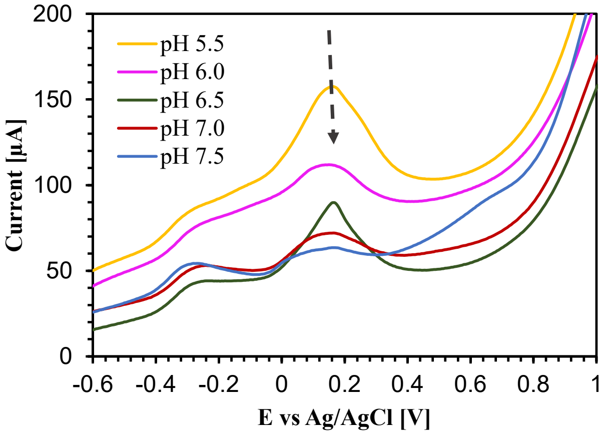

Figure S5. LSV curves of CuO−MWCNTs/SPCE electrode in 2 mM phosphate buffer (PB) pH from 5.5 to 7.5 and 0.1M KCl at scan rate 60 mV/s.

Author Contributions: Conceptualization, M.Y.A.; Data curation, M.Y.A and D.K.; Formal analysis, M.Y.A.; Investigation, M.Y.A and D.K; validation M.Y.A and D.K.; Writing - original draft, M.Y.A; writing—review and editing, M.Y.A., D.K. and M.M.R.H.; funding acquisition, M.M.R.H.; supervision, M.M.R.H. All authors have read and agreed to the published version of the manuscript.
